# Supplementary material for: Nutritional Niches of Cancer Therapy-Induced Senescent Cells
Source: Nutrients. 2022 Sep 2;14(17):3636. doi: 10.3390/nu14173636 (PMC9460569; doi:10.3390/nu14173636)
Supplement: Supplementary file 1 [file nutrients-14-03636-s001.zip › nutrients-1877761-supplementary.pdf]

SUPPLEMENTARY INFORMATION

Table S1. Raw dye reduction mean values (590 nm) of metabolite array from the PM-M1 microplate.

	CONTROL	ALISERTIB	DOXORUBICIN	CONTROL	BLEOMYCIN	PALBOCICLIB
**negative control**	0,164	0,228	0,261	0,159	0,262	0,245
**negative control**	0,146	0,227	0,264	0,150	0,283	0,250
**negative control**	0,143	0,243	0,272	0,148	0,274	0,231
**α-Cyclodextrin**	0,148	0,189	0,255	0,158	0,229	0,207
**Dextrin**	1,817	1,895	1,751	1,252	1,660	1,823
**Glycogen**	2,138	2,047	1,738	2,244	2,182	1,780
**Maltitol**	0,159	0,194	0,226	0,159	0,210	0,182
**Maltotriose**	1,954	1,456	1,600	1,742	2,052	1,477
**D-Maltose**	3,014	1,931	2,668	2,946	2,614	2,078
**D-Trehalose**	0,182	0,228	0,280	0,205	0,275	0,209
**D-Cellobiose**	0,143	0,188	0,269	0,152	0,201	0,204
**β-Gentiobiose**	0,144	0,180	0,230	0,148	0,213	0,173
**D-Glucose-6-P**	0,386	0,463	0,635	0,324	0,756	0,278
**α-D-Glucose-1-P**	0,545	0,415	0,977	0,169	0,470	0,578
**L-Glucose**	0,152	0,203	0,353	0,215	0,284	0,222
**α-D-Glucose**	3,068	1,980	2,586	2,957	2,777	2,127
**α-D-Glucose**	3,051	2,078	2,732	3,012	2,618	2,195
**α-D-Glucose**	2,941	2,322	2,529	3,045	2,829	1,975
**3-O-Methyl-D-Glucoside**	0,195	0,220	0,404	0,209	0,271	0,183
**α-Methyl-D-Glucoside**	0,151	0,202	0,393	0,179	0,266	0,207
**β-Methyl-D-Glucoside**	0,153	0,211	0,412	0,202	0,256	0,223
**D-Salicin**	0,228	0,265	0,482	0,235	0,329	0,227
**D-Sorbitol**	0,149	0,199	0,410	0,162	0,244	0,223

**N-Acetyl-D-Glucosamine**	0,143	0,193	0,293	0,155	0,237	0,184
**D-Glucosaminic Acid**	0,144	0,267	0,315	0,149	0,291	0,262
**D-Glucuronic Acid**	0,153	0,203	0,382	0,177	0,299	0,229
**Chondroitin-6-Sulfate**	0,203	0,263	0,381	0,268	0,355	0,266
**Mannan**	0,218	0,273	0,419	0,291	0,338	0,227
**D-Mannose**	2,648	1,486	1,908	2,532	2,237	1,764
**α-Methyl-D-Mannoside**	0,154	0,246	0,365	0,264	0,293	0,227
**D-Mannitol**	0,140	0,201	0,334	0,261	0,269	0,215
**N-Acetyl-β-D-Mannosamine**	0,154	0,210	0,387	0,260	0,268	0,221
**D-Melezitose**	0,190	0,220	0,360	0,268	0,273	0,239
**Sucrose**	0,195	0,231	0,402	0,275	0,269	0,227
**Palatinose**	0,161	0,217	0,375	0,178	0,253	0,241
**D-Turanose**	0,238	0,306	0,408	0,248	0,448	0,263
**D-Tagatose**	0,168	0,236	0,271	0,178	0,282	0,214
**L-Sorbose**	0,166	0,238	0,366	0,229	0,314	0,251
**L-Rhamnose**	0,185	0,259	0,334	0,264	0,302	0,230
**L-Fucose**	0,217	0,224	0,349	0,257	0,285	0,234
**D-Fucose**	0,238	0,249	0,326	0,308	0,322	0,251
**D-Fructose-6-P**	0,403	0,500	0,889	0,459	0,656	0,464
**D-Fructose**	0,240	0,243	0,422	0,299	0,408	0,254
**Stachyose**	0,184	0,219	0,357	0,238	0,274	0,223
**D-Raffinose**	0,203	0,207	0,357	0,250	0,272	0,255
**D-Lactiol**	0,204	0,218	0,370	0,267	0,292	0,238
**Lactulose**	0,163	0,213	0,370	0,186	0,257	0,224
**α-D-Lactose**	0,145	0,195	0,257	0,151	0,233	0,180
**Melibionic Acid**	0,154	0,231	0,282	0,165	0,300	0,239

**D-Melibiose**	0,159	0,214	0,390	0,170	0,277	0,243
**D-Galactose**	0,441	0,297	0,519	0,482	0,639	0,389
**α-Methyl-D-Galactoside**	0,201	0,219	0,346	0,260	0,287	0,244
**β-Methyl-D-Galactoside**	0,223	0,227	0,379	0,289	0,276	0,245
**N-Acetyl-Neuraminic Acid**	0,198	0,243	0,342	0,248	0,294	0,249
**Pectin**	0,208	0,232	0,334	0,268	0,261	0,216
**Sedoheptulosan**	0,175	0,225	0,375	0,265	0,285	0,243
**Thymidine**	0,211	0,226	0,378	0,278	0,260	0,253
**Uridine**	0,258	0,267	0,538	0,323	0,487	0,345
**Adenosine**	0,222	0,257	0,471	0,226	0,461	0,281
**Inosine**	0,211	0,326	0,585	0,233	0,645	0,410
**Adonitol**	0,162	0,235	0,280	0,155	0,254	0,225
**L-Arabinose**	0,243	0,255	0,411	0,223	0,364	0,282
**D-Arabinose**	0,244	0,236	0,337	0,278	0,275	0,261
**β-Methyl-D-Xylopyranoside**	0,213	0,238	0,356	0,263	0,283	0,248
**Xylitol**	0,222	0,235	0,387	0,291	0,341	0,259
**Myo-Inositol**	0,198	0,230	0,331	0,251	0,301	0,246
**Meso-Erythritol**	0,204	0,234	0,362	0,251	0,274	0,256
**Propylene glycol**	0,215	0,253	0,372	0,256	0,306	0,253
**Etanolamine**	0,239	0,176	0,341	0,278	0,214	0,192
**D,L-α-Gycerol-P**	0,223	0,327	0,605	0,302	0,326	0,368
**Glycerol**	0,146	0,203	0,377	0,162	0,257	0,203
**Citril acid**	0,145	0,204	0,220	0,159	0,298	0,161
**Tricarballylic Acid**	0,150	0,229	0,294	0,155	0,302	0,295
**D,L-Lactic Acid**	0,187	0,259	0,393	0,192	0,336	0,276
**Methyl D-Lactate**	0,145	0,223	0,356	0,164	0,309	0,274

**Methyl pyruvate**	0,144	0,251	0,361	0,171	0,338	0,268
**Pyruvic acid**	0,371	0,558	0,662	0,315	0,648	0,624
**α-Keto-Glutaric Acid**	0,229	0,269	0,457	0,210	0,487	0,312
**Succinamic Acid**	0,243	0,265	0,555	0,255	0,411	0,328
**Succinic Acid**	0,152	0,209	0,351	0,170	0,294	0,233
**Mono-Methyl Succinate**	0,216	0,283	0,640	0,233	0,456	0,363
**L-Malic Acid**	0,150	0,205	0,401	0,164	0,261	0,240
**D-Malic Acid**	0,147	0,220	0,323	0,154	0,253	0,229
**Meso-Tartaric Acid**	0,140	0,198	0,246	0,150	0,214	0,172
**Acetoacetic Acid (a)**	0,144	0,217	0,215	0,161	0,203	0,192
**γ-Amino-N-Butyric Acid**	0,147	0,244	0,299	0,138	0,287	0,253
**α-Keto-Butyric Acid**	0,148	0,259	0,303	0,153	0,277	0,259
**α-Hydroxy-Butyric Acid**	0,162	0,240	0,289	0,155	0,257	0,234
**D,L-β-Hydroxy-Butyric Acid**	0,149	0,216	0,296	0,149	0,250	0,205
**Glycolic Acid**	0,138	0,200	0,311	0,144	0,250	0,196
**Butyric Acid**	0,189	0,212	0,250	0,173	0,298	0,204
**2,3-Butanediol**	0,139	0,222	0,256	0,135	0,274	0,203
**3-Hydroxy-2-Butanone**	0,139	0,220	0,264	0,139	0,250	0,203
**Propionic Acid**	0,169	0,207	0,328	0,157	0,202	0,182
**Acetic Acid**	0,144	0,204	0,256	0,144	0,223	0,168
**Hexanoic Acid**	0,138	0,166	0,220	0,147	0,242	0,181

Table S2. Raw dye reduction mean values (590 nm) of metabolite array from the PM-M2 microplate.

	CONTROL	ALISERTIB	DOXORUBICIN	CONTROL	BLEOMYCIN	PALBOCICLIB
**negative control**	0,170	0,204	0,312	0,146	0,280	0,185
**negative control**	0,180	0,234	0,345	0,175	0,276	0,185
**negative control**	0,188	0,240	0,356	0,178	0,232	0,173
**Tween 20**	0,150	0,193	0,242	0,150	0,214	0,122
**Tween 40**	0,166	0,186	0,261	0,149	0,170	0,131
**Tween 80**	0,140	0,166	0,269	0,140	0,137	0,104
**Gelatin**	0,204	0,260	0,441	0,204	0,326	0,177
**L-Alaninamide**	0,180	0,278	0,43	0,187	0,218	0,175
**L-Alanine**	0,183	0,256	0,398	0,188	0,271	0,178
**D-Alanine**	0,197	0,235	0,336	0,189	0,257	0,173
**L-Arginine**	0,195	0,217	0,338	0,185	0,240	0,250
**L-Aspargine**	0,168	0,198	0,287	0,147	0,282	0,252
**L-Aspartic Acid**	0,192	0,243	0,34	0,203	0,241	0,166
**D-Aspartic Acid**	0,185	0,212	0,286	0,189	0,208	0,170
**L-Glutamic Acid**	0,266	0,291	0,476	0,268	0,310	0,254
**D-Glutamic Acid**	0,222	0,292	0,438	0,201	0,256	0,200
**L-Glutamine**	0,488	0,412	0,832	0,430	0,574	0,430
**Glycine**	0,203	0,280	0,455	0,192	0,228	0,232
**L-Histidine**	0,176	0,234	0,348	0,183	0,211	0,222
**L-Homoserine**	0,179	0,238	0,326	0,195	0,202	0,226
**Hydroxy-L-Proline**	0,178	0,238	0,304	0,189	0,218	0,198
**L-Isoleucine**	0,210	0,248	0,32	0,217	0,244	0,168
**L-Leucine**	0,205	0,244	0,313	0,205	0,259	0,181

**L-Lysine**	0,177	0,267	0,273	0,169	0,215	0,199
**L-Methionine**	0,206	0,271	0,372	0,226	0,232	0,183
**L-Ornithine**	0,211	0,266	0,472	0,200	0,269	0,231
**L-Phenylalanine**	0,309	0,293	0,405	0,281	0,273	0,281
**L-Proline**	0,255	0,263	0,32	0,259	0,240	0,196
**L-Serine**	0,266	0,239	0,335	0,245	0,226	0,247
**D-Serine**	0,247	0,266	0,385	0,254	0,228	0,278
**L-Threonine**	0,244	0,251	0,337	0,254	0,237	0,234
**D-Threonine**	0,261	0,242	0,322	0,251	0,213	0,234
**L-Tryptofan**	0,245	0,224	0,239	0,252	0,162	0,201
**L-Tyrosine**	0,276	0,262	0,245	0,279	0,166	0,202
**L-Valine**	0,219	0,247	0,318	0,212	0,265	0,235
**Ala-Ala**	0,190	0,304	0,431	0,178	0,276	0,327
**Ala-Arg**	0,193	0,335	0,491	0,208	0,290	0,315
**Ala-Asn**	0,222	0,279	0,464	0,220	0,244	0,300
**Ala-Asp**	0,249	0,272	0,377	0,268	0,269	0,269
**Ala-Glu**	0,260	0,421	0,628	0,273	0,311	0,421
**Ala-Gln**	0,500	0,671	0,907	0,450	0,584	0,630
**Ala-Gly**	0,254	0,288	0,399	0,248	0,238	0,293
**Ala-His**	0,259	0,251	0,329	0,228	0,284	0,250
**Ala-Ile**	0,309	0,308	0,382	0,286	0,261	0,286
**Ala-Leu**	0,277	0,285	0,321	0,230	0,301	0,272
**Ala-Lys**	0,230	0,297	0,417	0,239	0,269	0,251
**Ala-Met**	0,220	0,321	0,334	0,216	0,265	0,294
**Ala-Phe**	0,213	0,381	0,391	0,192	0,273	0,304
**Ala-Pro**	0,209	0,312	0,559	0,209	0,290	0,305

**Ala-Ser**	0,220	0,272	0,468	0,226	0,254	0,288
**Ala-Thr**	0,294	0,281	0,446	0,271	0,258	0,296
**Ala-Trp**	0,284	0,260	0,334	0,256	0,201	0,303
**Ala-Tyr**	0,295	0,325	0,519	0,281	0,312	0,304
**Ala-Val**	0,276	0,303	0,429	0,260	0,283	0,327
**Arg-Ala (b)**	0,279	0,315	0,432	0,240	0,344	0,310
**Arg-Arg (b)**	0,279	0,315	0,358	0,242	0,260	0,289
**Arg-Asp**	0,269	0,291	0,371	0,230	0,357	0,271
**Arg-Gln**	0,418	0,927	1,038	0,398	0,689	0,622
**Arg-Glu**	0,180	0,348	0,462	0,206	0,328	0,319
**Arg-Ile (b)**	0,181	0,389	0,402	0,178	0,313	0,260
**Arg-Leu (b)**	0,199	0,336	0,494	0,204	0,246	0,293
**Arg-Lys (b)**	0,201	0,287	0,495	0,211	0,257	0,316
**Arg-Met (b)**	0,296	0,289	0,406	0,259	0,252	0,327
**Arg-Phe (b)**	0,296	0,349	0,495	0,279	0,301	0,341
**Arg-Ser (b)**	0,266	0,309	0,433	0,252	0,331	0,277
**Arg-Trp**	0,235	0,242	0,303	0,234	0,223	0,266
**Arg-Tyr (b)**	0,266	0,318	0,484	0,261	0,371	0,304
**Arg-Val (b)**	0,275	0,317	0,451	0,241	0,300	0,305
**Asn-Glu**	0,283	0,332	0,463	0,250	0,426	0,300
**Asn-Val**	0,260	0,299	0,4	0,251	0,320	0,316
**Asp-Ala**	0,185	0,335	0,411	0,186	0,291	0,277
**Asp-Asp**	0,170	0,330	0,321	0,163	0,264	0,249
**Asp-Glu**	0,194	0,291	0,506	0,199	0,261	0,304
**Asp-Gln**	0,228	0,435	0,975	0,232	0,550	0,518
**Asp-Gly**	0,200	0,276	0,454	0,223	0,299	0,289

**Asp-Leu**	0,196	0,271	0,437	0,227	0,277	0,304
**Asp-Lys**	0,196	0,293	0,469	0,227	0,301	0,264
**Asp-Phe**	0,200	0,306	0,465	0,230	0,296	0,279
**Asp-Trp**	0,180	0,271	0,405	0,211	0,377	0,290
**Asp-Val**	0,179	0,279	0,381	0,214	0,297	0,274
**Glu-Ala**	0,213	0,405	0,607	0,215	0,387	0,317
**Glu-Asp**	0,197	0,323	0,431	0,202	0,300	0,256
**Glu-Glu**	0,204	0,340	0,504	0,204	0,341	0,332
**Gly-Gly**	0,221	0,331	0,488	0,190	0,258	0,462
**Glu-Ser**	0,199	0,290	0,512	0,206	0,267	0,289
**Glu-Trp**	0,204	0,245	0,339	0,225	0,178	0,361
**Gly-Tyr**	0,203	0,301	0,478	0,227	0,270	0,341
**Glu-Val**	0,216	0,304	0,598	0,210	0,268	0,389
**Gln-Glu**	0,263	0,718	1,11	0,297	0,683	0,714
**Gln-Gln**	0,403	0,690	1,107	0,410	0,631	0,752
**Gln-Gly**	0,328	0,588	0,983	0,354	0,617	0,597
**Gly-Ala**	0,197	0,315	0,411	0,193	0,259	0,283
**Gly-Arg**	0,203	0,273	0,359	0,192	0,245	0,245
**Gly-Asn**	0,196	0,316	0,404	0,191	0,266	0,271
**Gly-Asp**	0,194	0,326	0,347	0,186	0,244	0,270
**α-D-Glucose**	2,647	1,859	2,572	2,390	2,691	1,851

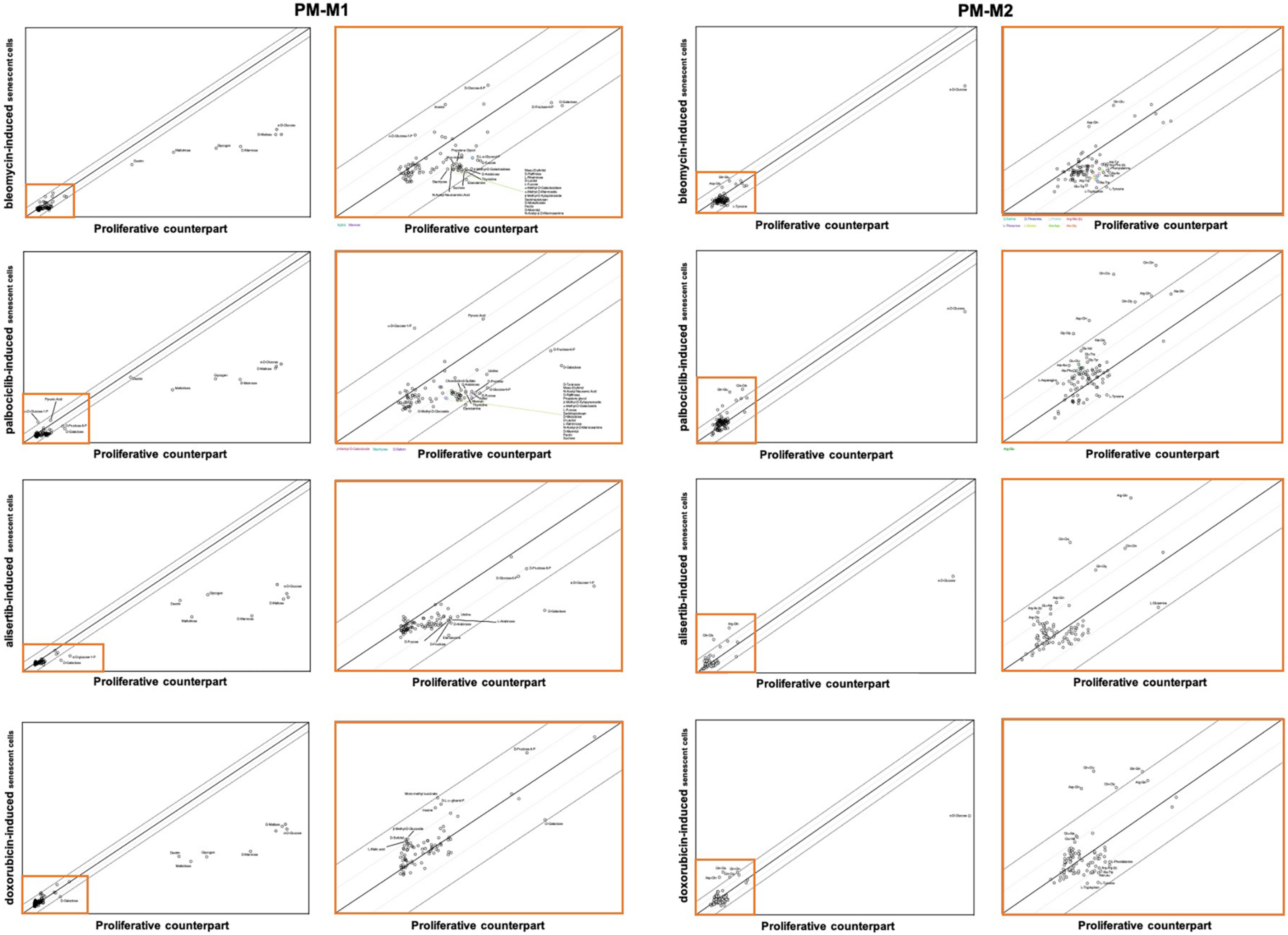
PM-M1
PM-M2
bleomycin-induced senescent cells
palbociclib-induced senescent cells
alisertib-induced senescent cells
doxorubicin-induced senescent cells
Proliferative counterpart

Figure S1. Comparison of substrate utilization by TIS and proliferative cells. To quantitatively compare each senescent state rapidly and systematically, changes in the substrate consumption of carbohydrate/carboxylate substrates (PM-M1) and amino acids/dipeptide combinations (PM-M2) were initially characterized based on the fold-change in the optical density of each substrate at 590 nm (purple color) resulting from the accumulation of reduced dye over a 90-minute period after normalization of the values to those of the negative-control wells included in each of the PM-M plates. We then calculated a comparison score from the absolute ratio between the substrate utilization of each pair of TIS cells and proliferative counterparts using ±1.5 (*grey dashed line*) and ±2.0 (*black dashed line*) fold-change cut-offs.

Proliferative counterpart #1

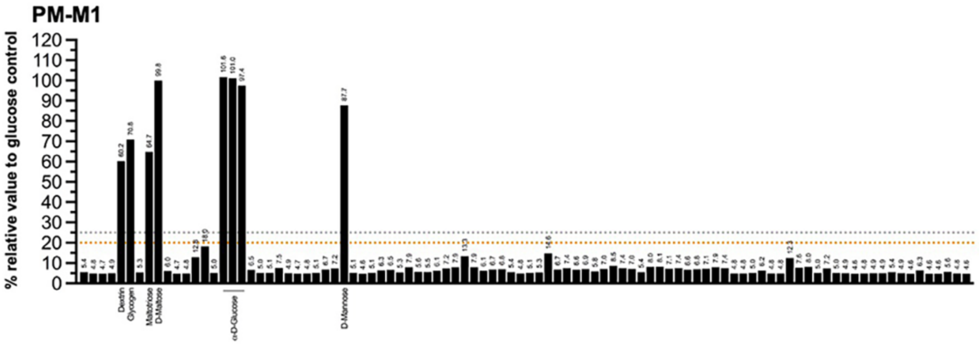

Proliferative counterpart #2

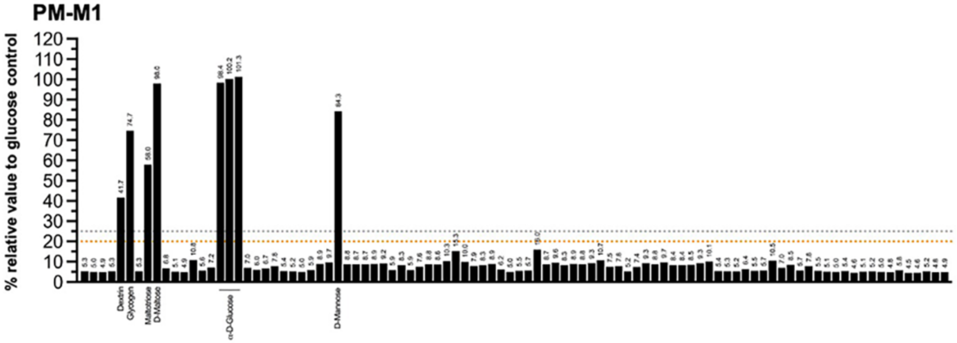

bleomycin-induced senescent cells

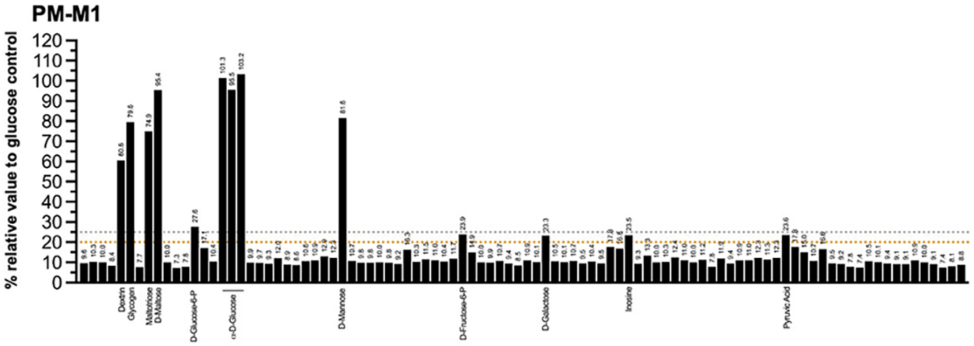

palbociclib-induced senescent cells

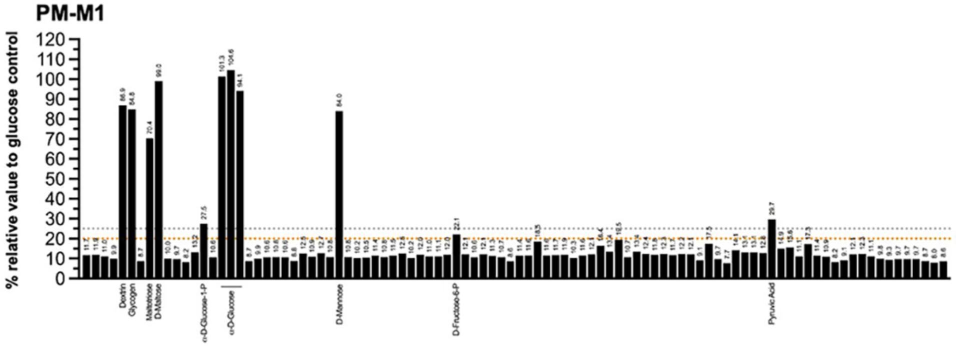

alisertib-induced senescent cells

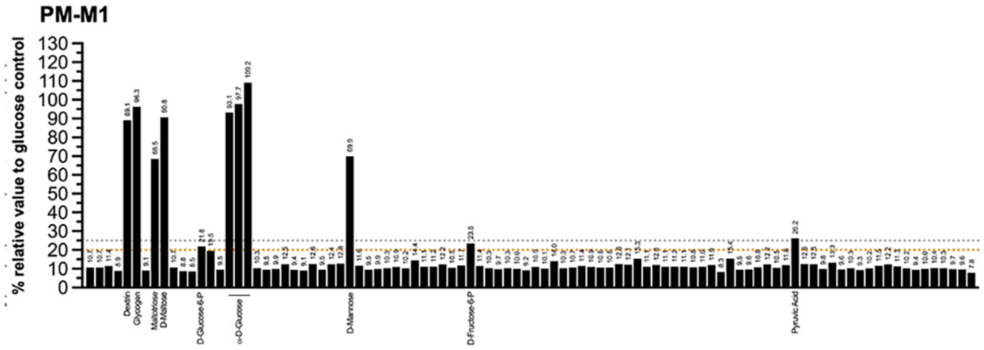

doxorubicin-induced senescent cells

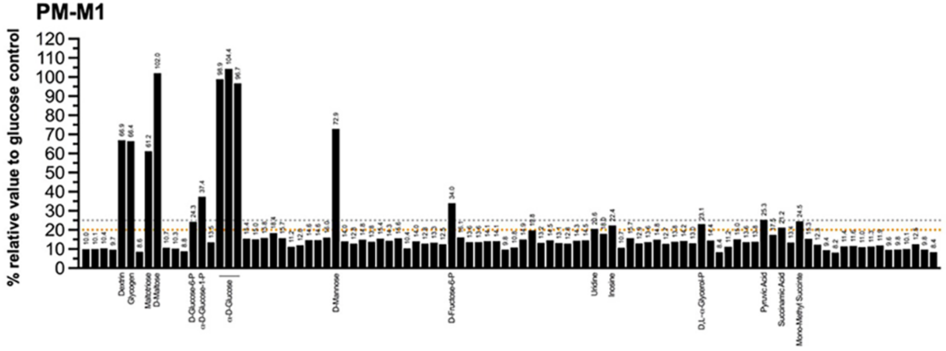

Proliferative counterpart #1

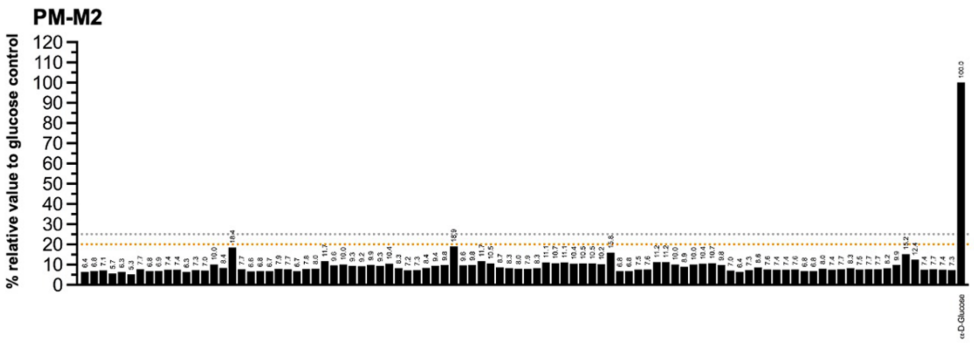

Proliferative counterpart #2

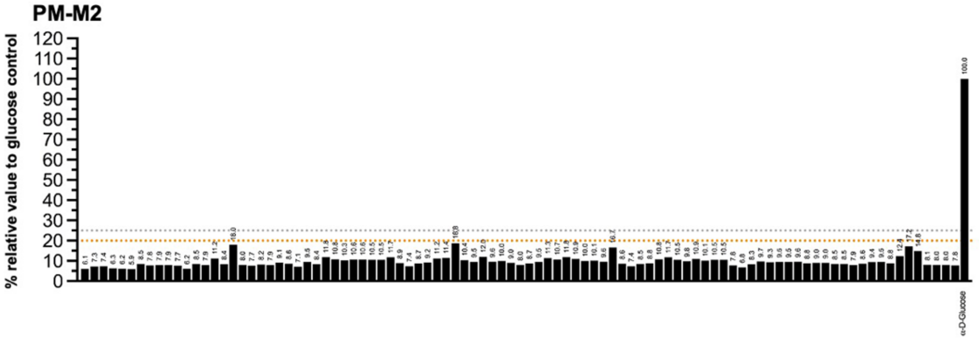

bleomycin-induced senescent cells

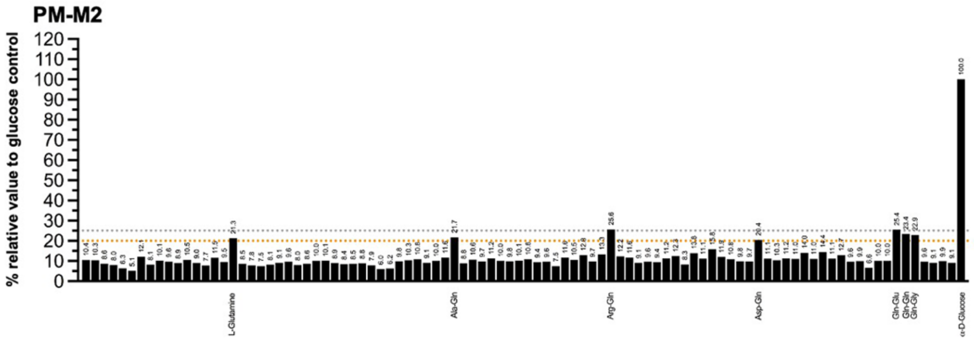

palbociclib-induced senescent cells

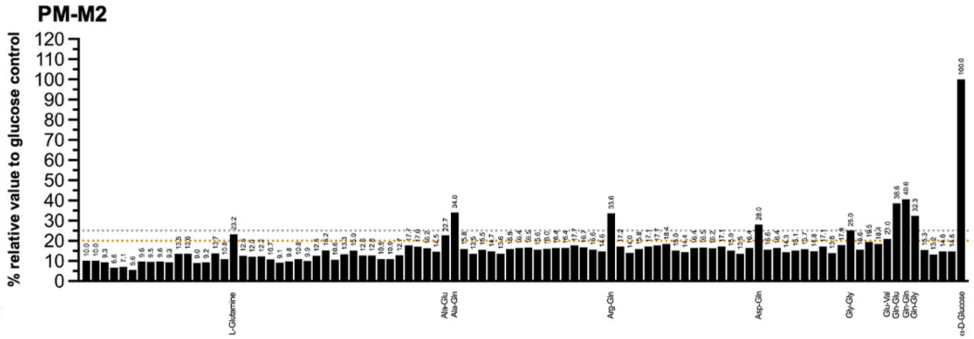

alisertib-induced senescent cells

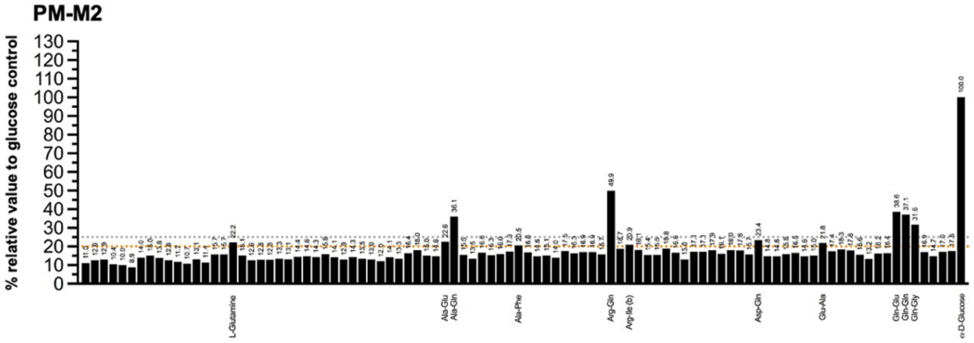

doxorubicin-induced senescent cells

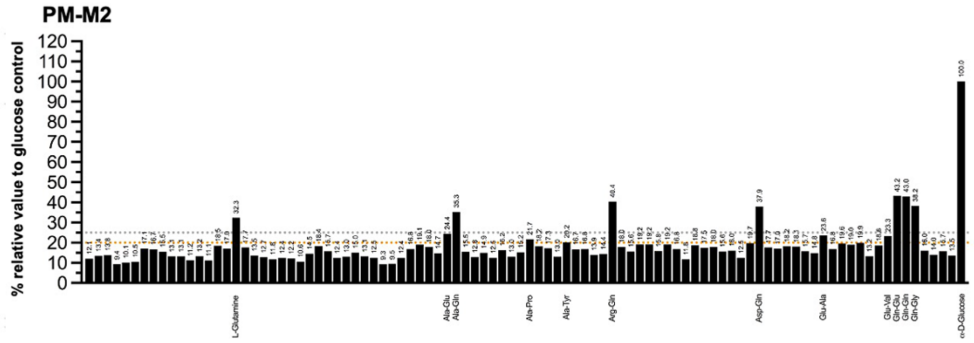

Figure S2. Metabolic fingerprints of nutrients supporting bioenergetic activity of TIS cells in the absence of glucose. Substrate utilization of proliferative/TIS cells was normalized to that of the positive controls, i.e., wells pre-loaded with glucose, in PM-M1 and PM-M2 plates. We established 20% (orange dashed line) and 25% (dark grey dashed line) cut-offs to select significant nutritional rescuers of glucose deprivation in TIS cells.
